# Mass Spectrometry Imaging with Trapped Ion Mobility Spectrometry Enables Spatially Resolved Chondroitin, Dermatan, and Hyaluronan Glycosaminoglycan Oligosaccharide Analysis *In Situ*

**DOI:** 10.1021/acs.analchem.4c02706

**Published:** 2024-10-30

**Authors:** Anthony Devlin, Felicia Green, Zoltan Takats

**Affiliations:** † The Rosalind Franklin Institute, Harwell Campus, Didcot OX11 0FA, U.K.; ‡ Faculty of Medicine, Department of Metabolism, Digestion and Reproduction, Imperial College London, South Kensington Campus, London SW7 2AZ, U.K.

## Abstract

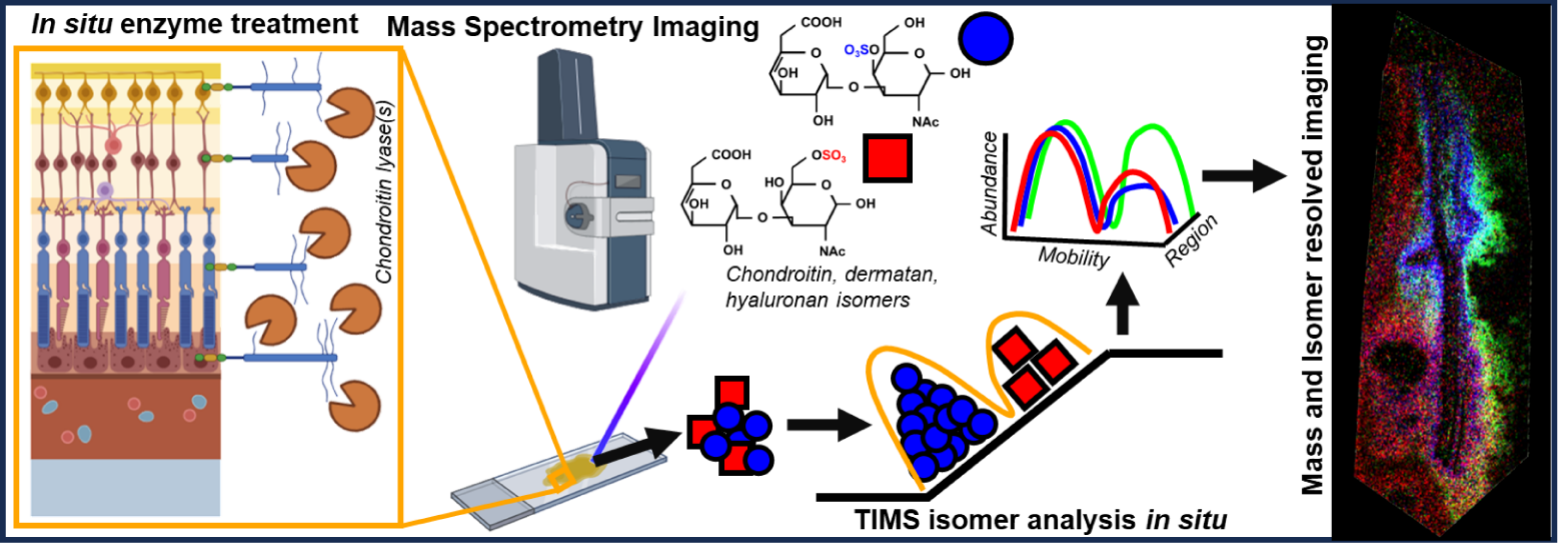

Previously, spatially resolved analysis of glycosaminoglycans (GAGs), by type and sulfation state, was unobtainable. Here, we describe a mass spectrometry imaging (MSI) approach which enables the detection, identification, localization, and profiling of GAG oligosaccharides directly from retinal tissue. Through *in situ* treatment of tissues with relevant chondroitinase enzymes, we liberate and spatially resolve chondroitin, dermatan, and hyaluronan from disaccharides through to hexasaccharides, directly from tissue sections. We demonstrate the separation of isomeric GAG oligosaccharide ions at different histologically relevant regions using trapped ion mobility spectrometry (TIMS). This paper describes the first spatially resolved analysis of multiple GAGs and their oligosaccharide sulfation state(s) directly from tissues.

Glycosaminoglycans (GAGs) are ubiquitous, vital polysaccharides^1^ which possess numerous important roles, ranging from tissue structure and stability^2,3^ to cell signaling,^4,5^ which in turn modulates numerous biological processes including cell growth and proliferation,^6^ cell adhesion,^7^ wound repair,^8,9^ and pathogenic invasion.^10−13^ GAGs possess complex structures which are characterized by, with the exception of hyaluronic acid (HA), levels and types of sulfation. GAGs also have a high pharmaceutical potential,^13−17^ which is likely due to their promiscuous binding activity, which is driven by sulfate composition.^18^ Despite their importance in human health and disease and the treatment thereof, little is known about the states of natural GAGs within the local cellular environment.^19^

Mass spectrometry imaging (MSI) is a well-established technique which enables the profiling of various molecules in a spatially resolved manner.^20^ To date, no direct attempt at analyzing the sulfation pattern of GAG oligosaccharides with MSI has been reported. Native fragments, attributed to sulfated HexNAc-HexA (N-acetyl hexosamine-Hexuronic acid) polymers which may correspond to GAGs have been detected with MALDI-FTICR-MSI^21^ (matrix assisted laser desorption ionisation-Fourier transform ion cyclotron resonance) but were not characterized further. Clift et al.^22^ utilized chondroitinase (CHase) digestion to improve downstream protease and PNGase F treatments, which enabled them to detect di- and tetra-saccharides (degree of polymerization; DP2 and 4) of chondroitin sulfate (CS). HA has also been imaged in human skin using hyaluronidase (H1136) digestion.^23^ No MSI methodologies have been reported for the analysis of sulfate composition, most likely because the majority of sulfated of di- and oligosaccharides are isomers.

Usually, the sulfate composition of GAGs is analyzed using in-line separation (either chromatographic or electrophoretic).^24^ In-line separation per pixel – while possible – is difficult to achieve, owing to long run times (minutes to hours per pixel depending on the analyte studied).^25^ Here, ion mobility spectrometry (IMS), a technique which separates ions based on their different collisional cross sections (CCSs), more colloquially, by their apparent size and shape in 3D space^26^ is utilized instead. Trapped IMS (TIMS), a variant of IMS developed by Bruker,^27^ offers improved resolving power and ion transmission in a small footprint compared to other IMS techniques^28^ and has separated derivatized heparin/heparan sulfate (HS) disaccharides^29^ and, CS and heparan sulfate DP4 and DP6 isomers previously.^30^

Here, we demonstrate the ionization, identification, and analysis of mass, mobility, and spatially resolved CS, the sister molecule of CS, dermatan sulfate (DS), and HA ions generated through *in situ* tissue digests.

## Methods

### GAG Standards

CS disaccharide (CD002-5) and oligosaccharide (CS004-6), and HA (HA004-6) standards were acquired from Iduron (Manchester, UK). CS oligosaccharide standards were produced by the CHase ACI (CHase AC) digestion of shark cartilage. CHase AC and CHase B were also purchased from Iduron.

### Nomenclature

**Figure 1:**
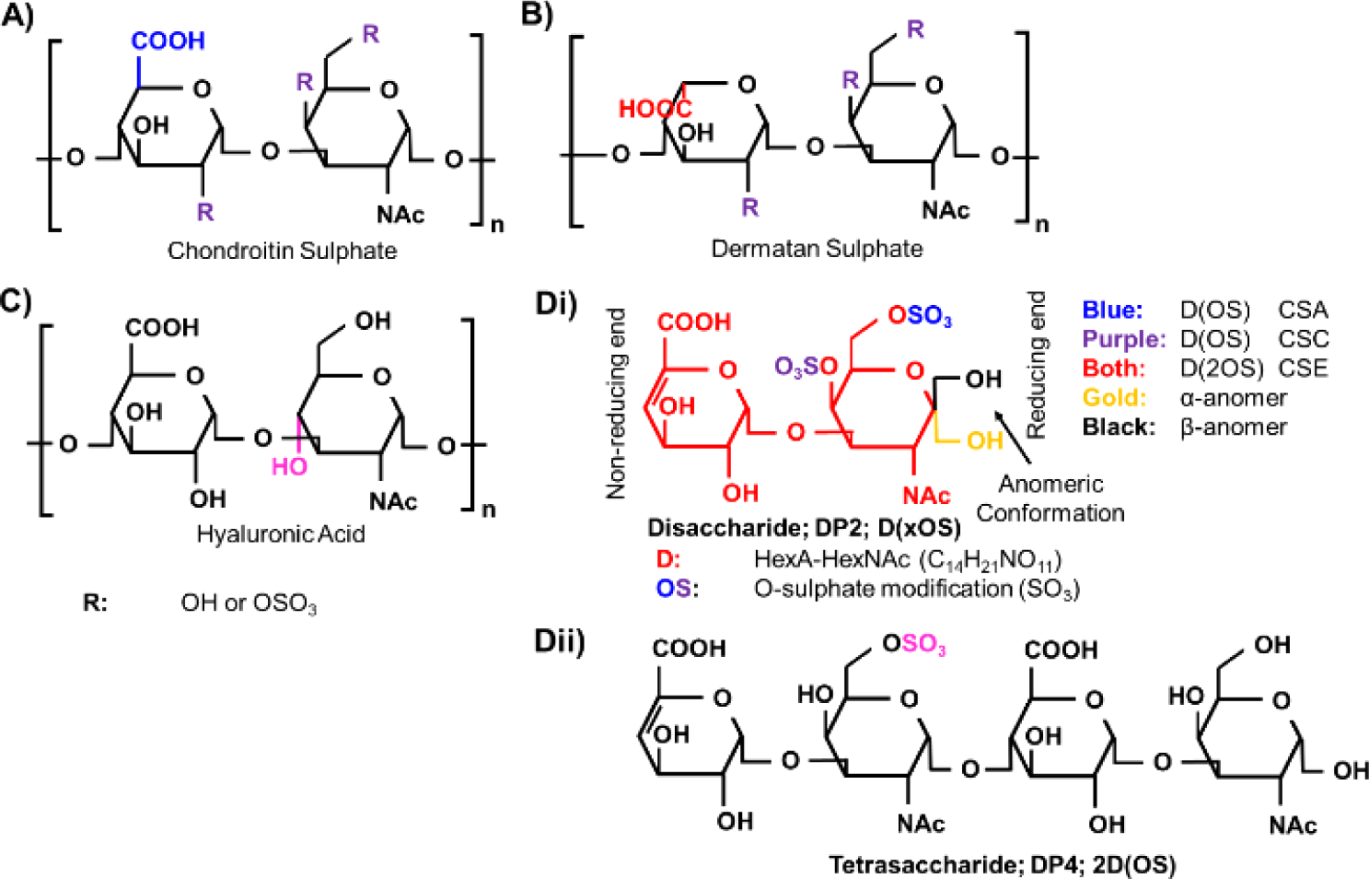
Chondroitin, dermatan, and hyaluronan structures. A) CS (GlcA-GalNAc; Glcuronic acid-Galactosamine) structure. B) DS (IdoA-GalNAc; iduronic acid) structure. C) HA (GlcA-GlcNAc; glucosamine) structure. D) Examples of the structures and their corresponding ion nomenclature. i) examples of disaccharides (D(xOS)). For D(xOS), D indicates a single disaccharide of mass C_14_H_21_NO_11_ (red and gold or black), while a single OS indicates the addition of an SO_3_ by mass (blue or purple). A chondroitin disaccharide containing only one sulfate at C-4 of GlcNAc (purple) is a CSA disaccharide and at C-6 of GalNAc (blue) is a CSC disaccharide, and that containing two at both C-4 and C-6 is a CSE disaccharide. Other CS disaccharides exist. The reducing end forms an equilibrium of different anomers in solution: α- (gold), β- (black), and open chain (not shown here); ii) example of a singly sulfated tetrasaccharide: 2D(OS).

We describe ions based on disaccharide and sulfate composition, where D indicates a single, unsulfated, unsaturated HexA-HexNAc disaccharide (C_14_H_21_NO_11_). OS indicates an O-sulfate modification and corresponds to the addition of SO_3_ with regard to mass (Figure 1Di). Consequently, a singly sulfated disaccharide is represented as D(OS) (Figure 1Di), while a tetrasaccharide with one sulfate is represented as 2D(1OS) (Figure 1Dii). Generic chain lengths regardless of the sulfation state are referred to by their degree of polymerization (DPX), where X is the number of saccharide residues; e.g., a hexasaccharide (6-mer) is a DP6. The number of sulfates per disaccharide residue (degree of substitution; DOS) can be easily calculated where, for XD(NOS), the DOS is N/X. For fragments, Domon and Costello nomenclature^31^ is utilized.

### Tissue Sourcing/Sectioning/Mounting

Retinae sections were from mature cynomolgus primates of Indonesian origin and were a kind gift from Glen Jeffrey (Institute of Ophthalmology, University College London, UK). The animals were culled as part of a process of reducing the size of a colony at Public Health England, UK. Animals were deeply anesthetized, and at the point of death as determined by the cessation of breathing and heartbeat, the eyes were removed . The eyes were then immersed in 4% paraformaldehyde for 24 h, washed in phosphate buffer, and stored in phosphate buffer with 1% sodium azide at 4 °C. Prior to processing, the anterior chamber and lens were removed, and targeted regions of the eye cup were immersed in 30% sucrose in phosphate buffer for 24 h. These were then sectioned on a cryostat at 10 μm, mounted on Superfrost slides, and stored at −80 °C. Upon removal from the freezer, sections were immediately desiccated for at least 45 min before further preparation was undertaken. An enzyme (chondroitinase ABC (9024-13-9; Merck, Darmstadt, Germany), chondroitinase AC-I (CDAC-ENZ; Iduron, Alderley Park, UK), or chondroitinase B (CDB-ENZ; Iduron) at a concentration of 1 IU·ml^–1^ in 50 mM ammonium acetate (631-61-9; Merck) for CHase AC and ABC or 49 mM ammonium acetate, 1 mM Ca acetate (5743–26–0; Fluorochem, Glossop, UK) for CHase B) was applied to the tissue by a TM HTX3 (HTX Technologies LLC, Chapel Hill, NC, USA) sprayer using the standard trypsin parameters (nozzle temperature = 40 °C, gas pressure = 10 psi, flow rate = 10 μL/min, velocity = 1200 mm/min, track spacing = 3 mm, number of passes = 15 in a criss-cross pattern with 0 drying time), corresponding to approximately 30 μl (30mU) of enzyme per section. Sections were incubated in a pseudohumidity chamber as described in Angel et al.^32^ for 2 h (CHase ABC) or 5 h (CHase AC, B) at 37 °C before desiccation for at least 45 min prior to matrix application.

### Mass Spectrometry

MS analysis was undertaken using a tims-TOF fleX (Bruker Daltonics, Bremen, DE) instrument in negative ion mode. The MALDI matrix, 15 mg·ml^–1^ 9-aminoacridine (92817; Sigma-Aldrich, St. Louis, MO), 0.2% formic acid (Z0797502 21; Sigma-Aldrich) in 69.9% methanol (14262; Fisher Scientific, Hampton, NH), and 29.9% LC grade water (7732-18-5; Fisher Scientific) was used. The matrix was applied using a TM HTX3 sprayer (nozzle temperature = 80 °C, gas pressure = 10 psi, flow rate = 100 μL/min, velocity = 1200 mm/min, track spacing = 3 mm, number of passes = 16 in a criss-cross pattern with 2 s drying time). The instrument was mass and mobility calibrated using direct infusion ESI of the tune mix (G2431; Agilent Technologies, Santa Clara, CA, USA). The TIMS buffer gas flow was allowed to equilibrate for at least 30 min after loading the MALDI plate and then set to 2.85–2.87 mbar.

The ion source parameters were set as follows: for MALDI, MALDI offset = 50 V and shots = 200, rate = 10 kHz. For direct infusion ESI, an end plate offset of 500 V and capillary voltage of 3000 V were used, with the nebulizer set to 0.3 bar, the dry gas at 3.5 L/min, and the dry temperature at 200 °C. The instrument parameters were as follows (for a mass range of 50–2000 *m*/*z*, using a quadrupole mass filter at 200 *m*/*z* with 850 Vpp collision RF): deflection 1 delta = −70 V, funnel 1 RF = 500 Vpp, funnel 2 RF = 200 Vpp, multipole RF = 200 Vpp, isCID = 0v, ion energy = 5 V, CID = 5 V. The TOF transfer time was 110 μs, and the pre-pulse storage was 10 μs. TIMS was run with N_2_ as the buffer gas, and the parameters were the following: accumulation time = 20 ms (corresponding to 200 shots when using MALDI), ramp time = 200 ms, ramp start (1/k_0_start; voltage) = 0.60 V·s/cm^2^; 242.8 V, ramp end (1/k^_0_^end; voltage) = 1.80 V·s/cm^2^; 46.5 V. TIMS offsets set with IMEX were Δ*t*1 = 20 V, Δ*t*2 = 120 V, Δ*t*3 = −70 V, Δ*t*4 = −150 V, Δ*t*5 0 V, Δ*t*6 = −150 V, and collision cell in = −300 V.

Imaging parameters such as pixel size and x,y coordinates were set using Flex Imaging (Bruker Daltonics) software. The majority of images were acquired at a 20 μm pixel size, while one set of Chase AC images was acquired at a 40 μm pixel size.

MS/MS experiments were performed for ions with sufficient signal and sodiation to yield fragments (D(1-2OS), 2D(0-2OS), and 3D(0-2OS)) using CID (25–55 eV). Comparison of sample TIMS profiles to those of the CS standards was also used.

### Data Analysis

Imaging data sets were imported into the SCiLS Lab (Bruker) environment for image analysis. All images were normalized to the total ion count (TIC). For TIMS profile analysis, imaging data sets were opened in Compass data analysis (Bruker), where whole tissue sections or regions of interest were selected using the image window, and mobilograms were subsequently generated in that region for ions of interest. The mobilograms were exported as .xy files and imported into R for statistical analysis. Mobilities were normalized to maximum signal intensity (0–1). Mean centering and subsequent PCA of mobilograms were performed using the *prcomp* function in R. No other signal processing was applied. MS/MS fragments were compared to those generated from CS standards and assigned using GlycoWorkbench.^33^

### Histology

H&E staining was performed on sections after MALDI analysis using an Abcam (Cambridge, UK) H&E staining kit (ab245880) as per the manufacturer’s instructions with one small change: sections were first washed in 100% methanol to remove the matrix.

## Results and Discussion

### *In situ* Enzyme Digests Yield Ions That Correspond to Chondroitin, Dermatan, and Hyaluronan

**Figure 2:**
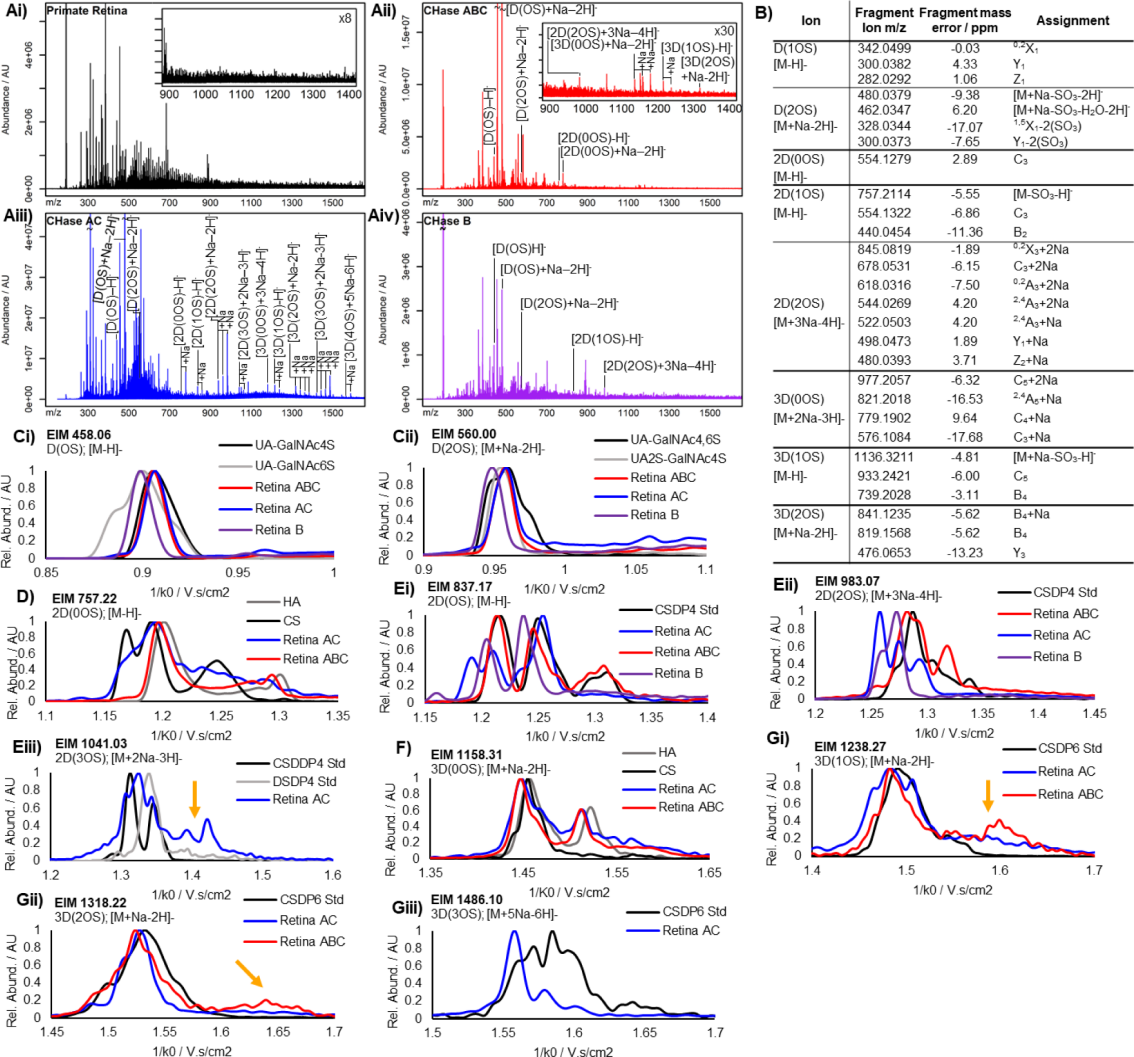
MALDI IMS-MS analysis of primate retina sections treated with different chondroitinase enzymes. A) Mass spectra of primate retina sections treated with (i) no enzyme; (ii) Chase ABC; (iii) CHase AC; (iv) CHase B. Ions that correspond to GAGs are labeled. B) CID fragments of ions of interest at 25–55 eV. C–G) Extracted ion mobilities (EIM) of DP2 unsulfated (C) and sulfated (D), DP4 unsulfated (E) and sulfated (F), and DP6 (G) ions detected in primate retinae (red, blue, purple: ABC, AC, B) compared to GAG standards (black, gray). Orange arrows indicate isobaric noise peaks due to a low ion signal. All mobilities are normalized (0–1). A full set of EIMs for each ion and comparisons to standards can be found in Figures S1–S4.

Direct MALDI analysis of tissue sections yielded no sulfated saccharide fragments as were observed by Kunzke et al.^21^ (Figure 2Ai). CHase ABC has previously been shown to yield disaccharides (DP2) and tetrasaccharides (DP4) from aortic valve sections.^22^ Therefore, CHase ABC was applied to primate retina tissue sections facilitating detection of ions correlated to DP2s with 1 and 2 sulfates, DP4s containing 0, 1, and 2 sulfates, and hexasaccharides (DP6) containing 0, 1, and 2 sulfates (Figure 2Aii). Primarily DP2s were yielded, with weak signals for DP4s and DP6s. To improve the yield of larger oligosaccharides, two other enzymes, CHase AC and CHase B, which have different substrate specificity, were used. CHase AC yielded the same DP2s but with lower intensity, and ions that correspond to DP4 and DP6s, with 0–3 and 0–4 sulfates, respectively at higher intensities (Figure 2Aiii). CHase B yielded a smaller subset of DP2 and DP4 ions with no unsulfated oligosaccharides detected (Figure 2Aiv). A full list of the detected and assigned ions that correlate with GAGs can be found in Table S1. MS/MS was carried out on ions of interest with a sufficient signal (D (1-2OS), 2D(0-2OS), and 3D(0-2OS)) using CID (25–55 eV) (Figure 2B) and were compared to MS/MS of CS standards from shark cartilage of the same mass to confirm ion identity. The remaining ions were assigned putatively, based on presence only after treatment with a CS-lyase enzyme and accurate mass.

IMS profiles of all ions detected from the primate retina after digestion were compared with those acquired from CS standards after digestion (Figures 2C–G and S1–S5). The mobilities, particularly those from CHase ABC-treated tissue, were found to have comparable values to those of the same mass from commercially available CS oligosaccharides from shark cartilage (standards) (Figure 2Ei,ii), facilitating confirmation in the identification of remaining ions as GAGs without MS/MS. While the mobility profiles appear comparable in peak location, some possessed different peak ratios and occasionally extra peaks, particularly in the CHase AC-treated samples.

Derivatized HS and CS oligosaccharides with different sequences have been shown to have unique mobilities^30^ hence, the differences observed between different mobility profiles are most likely due to differing abundance and/or presence of different oligosaccharide sequences in the samples. For DP2s, one or two unresolved peaks are observed, likely corresponding to the canonical CSA or CSC for D(OS) or the CSB and CSE for D(2OS) DP2s (Figure 2C). Multiple peaks are observed for unsulfated CS and HA DP4s, indicating that different conformations are present and separated with TIMS as no sulfate isomers are present (Figures 1Di, S5). Unique mobilities for both CS and HA are present, enabling the identification of both in situ (Figure 2D,F).

As the residue number increases, the number of possible sulfate combinations for a sulfated oligosaccharide increases. For a DP4 system containing only CSA, CSC, or unsulfated (0S) DP 2s, four possible combinations at the mass of 2D(OS) and four combinations at the mass of 2D(2OS) exist. For 2D(OS), B_3_ fragments that correspond to the mass of a GlcA-GlcNAc-GlcA+SO_3_ were yielded (Figure 2B), suggesting that the sulfate is present only on the nonreducing end disaccharide. Hence, only two possible combinations exist (CSA-CS0S or CSC–CS0S), which can be observed in the CHase AC treated mobilities (Figure 2Ei, blue). The split peaks here can likely be attributed to different anomer or ring conformers (Figure S5), as observed in unsulfated species. The detection of other peaks in shark cartilage DP4s, and CHase ABC and B treated sections suggest either the presence of rarer sulfates, such as CS-2OS and/or IdoA, which cannot be observed in DP2s as the HexA identity is lost during enzyme cleavage. Since both CHase ABC and CHase B target DS, which contains IdoA (unlike CHase AC), this is likely why extra oligosaccharide sequences are detected here.

Four peaks are observed in 2D(2OS) DP4s (Figure 2Eii) for both CHase ABC and AC-treated sections, which may correspond to the cannonical CSA-CSA, CSA-CSC, CSC–CSA, and CSC–CSC DP4s. No assignment of each structure was made to any particular peak. Multiple, overlapping mobilities are observed as DP6s are analyzed (Figure 2G). With 3D(3OS), a complex mobility profile is observed in shark cartilage DP6s (Figure 2Giii, black), containing up to 6 structures. This is contrasted against a relatively simple 3D(3OS) profile detected in primate retinae, suggesting a more homogeneous CS sequence across primate retinae.

Differences observed between different enzymes are likely due to differences in enzyme specificities, which will, therefore, yield different oligosaccharides at different rates. This is especially obvious when CHase AC and CHase B (Figure 2, blue vs purple)-treated mobilities are compared, as they primarily target different GAGs: CS and DS respectively. Tailing can be observed in the mobilities, particularly in Figure 2Gi. This is likely due to isobaric noise appearing in mobilities isolated from samples with a weaker signal. The effect of signal strength on tailing can be observed in Figure 2Gii, orange arrow, where the standard (black) has the strongest signal, followed by the CHase AC treated tissue (blue) and the CHase ABC treated tissue (red).

### Spatial Resolution of GAG Oligosaccharides

**Figure 3:**
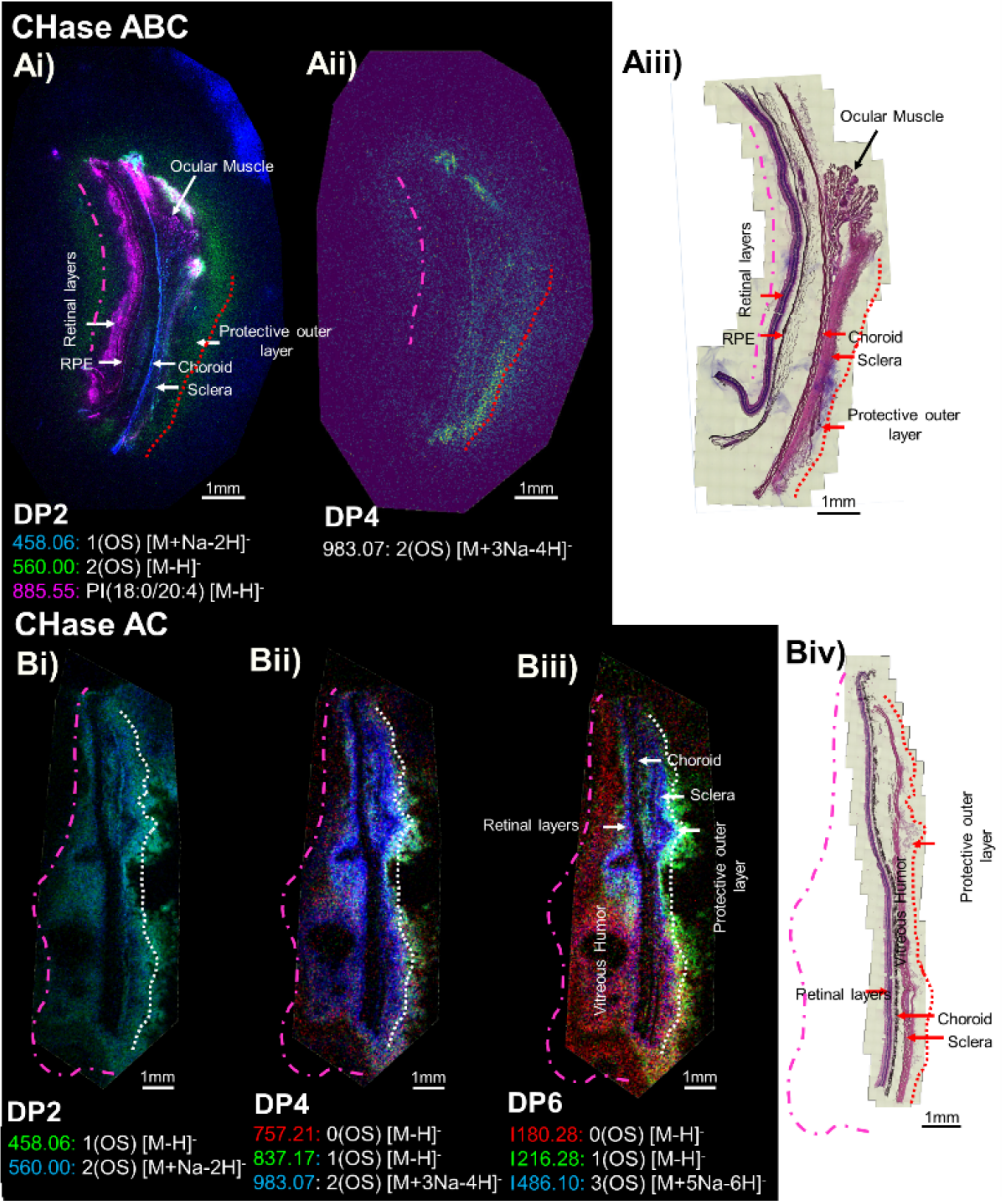
MALDI-TOF MS images of the GAG oligosaccharides. A) Stacked ion images of a primate retina after CHase ABC treatment shown for DP2 (i) and DP4 (ii), and (iii) the labeled H&E stained section. B) Ion images of a primate retina after CHase AC treatment shown for DP2 (i), DP4 (ii), and DP6 (iii) ions and (iv) the labeled H&E stained section. White/red dotted lines indicate the edge of the protective outer layer and the pink dashed line the edge of the vitreous humor, both of which do not stain well with H&E. Single ion images for all GAG ions can be found in Figures S7 and S9.

**Figure 4:**
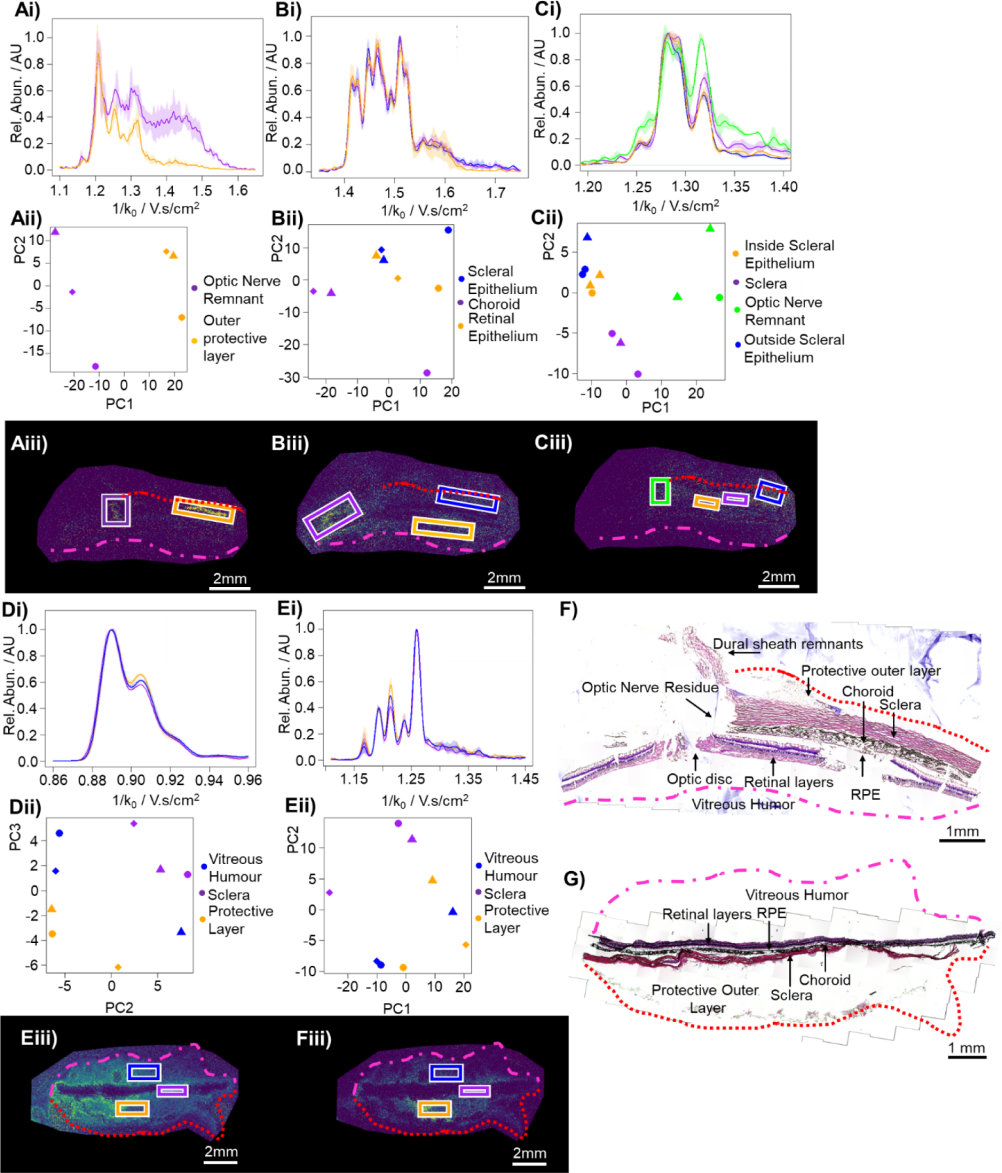
Spatially resolved analysis of oligosaccharide profiles from the primate retina. A,B,C) Analysis of 2D(OS), 3D(0OS), and 2D(2OS) respectively, liberated with CHase ABC. D,E) Analysis of D(OS) and 2D(1OS) respectively, liberated with CHase AC. These consist of i) average EIM of three repeats ± standard deviation. ii) PCA scores plot for PC1 vs PC2 or PC2 vs PC3 for the three repeats. iii) Ion images, boxes indicate the areas examined. Data were acquired across *n* = 3 serial sections. F,G) Labeled H&E stained optical image for the sections treated with CHase ABC and AC respectively. Red dotted lines and pink dashed lines indicate the edge of the protective outer layers and vitreous humor, respectively.

MALDI MSI analysis of the primate retina after enzyme digestion was able to spatially resolve the identified sulfated oligosaccharide ions (Figure 2) and is shown alongside the histological identification of the retinal layers (Figure 3). H&E staining struggles to highlight all the regions of the sections in which GAGs are present. The vitreous humor, as it is 99% water, is often washed away during staining and hence is not indicated histologically,^34^ however, optical images of the sections after matrix application indicate regions which likely correspond to the vitreous humor (Figure S6ii). The protective outer layer is also poorly stained, but the outside edge can be observed in H&Estained sections. This is most clear in later sections (Figure 4G). Extensions beyond these regions may have been due to diffusion during embedding or delocalization during enzyme treatment. The vitreous humor and outer protective layers are indicated in ion images where applicable with dashed and dotted lines respectively and were determined from optical images of the sections after either matrix application or H&E staining (Figure S6).

Disaccharides yielded from CHase ABC digestion were located across the entire section (Figure 3Ai; blue and green). D(OS) is localized within the retinal layers, choroid, and sclera (Figure 3Ai; blue), while D(2OS) is localized to only the choroid and sclera (Figure 3Ai; green) and also forms a halo around the outside of the retinal layers and sclera, which is likely part of the vitreous humor and outer protective layer. Low levels of DP4 (Figure S7B) – specifically 2D(2OS) – were also identified (Figure 3Aii), appearing with localization comparable to that of D(2OS). Other DP4 and DP6 saccharides were detected in situ in other comparable sections and could be localized but had generally poorer signals (Figure S8). When treated with CHase AC, D(OS) and D(2OS) appear diffuse and, unlike CHase ABC treatment, share considerable overlap (Figure 3B). Both locate to the sclera, protective outer layer, and into the vitreous humor.

As the residue number increases, i.e. from a DP2 to a DP4, the localization becomes more specific (Figure 3Aii,Bii) such that spatial differentiation can be observed for oligosaccharides with different degrees of sulfate substitution (DOS) (Figure 3Bii). When analyzing DP6s (Figure 3Biii), improved localization is observed with unsulfated DP6s (3D(0OS); red), while DP6s with DOS ≥ 1 (3D(3–4OS); blue) localize similarly to DP4s with the same DOS. The distinction between these two sulfate levels becomes more apparent in DP6s than that in DP4s, with less sulfated DP6s (0 < DOS < 1; 3D(1-2OS)) localizing to the edges of the different tissue layers, while the most sulfated DP6s localize to the tissue stroma.

The observation of a difference between the protective layer and the stroma can be made using either enzyme, either as a halo of DP2s with DOS > 1 when using CHase ABC (Figure 3A) or as a halo of oligosaccharides with DOS < 1 when using CHase AC (Figure 3Bii,iii green). This is important to note, as both demonstrate that these regions have different CS profiles, but the way in which this is observed is dependent on the specificity of the enzymes used to probe them. CHase ABC will digest both CS and DS, yielding GlcA- and IdoA-containing sequences, while CHase AC will digest primarily GlcA containing CS chains, yielding IdoA-containing oligosaccharides. This explains why the same distinct tissue regions are observed through highly or lowly sulfated saccharide sequences. Furthermore, unsulfated DP2s are not detected, even with standards, using this matrix system, thus, the lowly sulfated information observed in CHase AC oligosaccharides is lost when examining only disaccharides. To investigate the halo further, we treated another section with CHase B (which targets IdoA-containing sequences; DS). Essentially no DP2s or DP4s were yielded in the retinal layers, choroid, or sclera and the signal was found primarily in the vitreous humor and protective outer layer (Figure S10), corresponding to the D(2OS) and lowly sulfated CS halos observed when treated with CHase ABC and AC respectively, suggesting that this halo contains DS chains.

### TIMS Profiles Alter Depending on Spatial Location

Localization of GAGs based on DOS (i.e., mass-resolved ions) has been achieved, but the analysis of the type of sulfation has yet to be achieved. It has been demonstrated previously that TIMS profiles correlate to the relative abundance of different GAG oligosaccharides;^29,30^ therefore, we probed the ability of TIMS to analyze oligosaccharide composition at different locations *in situ* (Figure 4). Single-pixel TIMS profiles were noisy, hence the TIMS profiles averaged across regions of interest, which were selected based on a combination of signal strength and histology, were instead investigated. Irregular ROIs were unable to be drawn in Compass data analysis, so rectangles of similar size in the relevant regions were drawn, and the average mobilograms were compared to each other. Improvements in data extraction and signal processing will likely enable images based on mobility profiles to be generated in the future.

First, 2D(OS) was examined due to two clear localizations of signal at the protective outer layer and at the remnant of the optic nerve (Figure 4Aiii). The average mobility across both regions was extracted from three serial sections, and all mobilograms were subjected to principal components analysis (PCA) to determine similarity. These mobilities, while noisy, had different profiles (Figure 4Aii). The same trend was then observed for 3D(0S) (Figure 4B), where unsulfated DP6s at the outside edges of the nerve fiber layer and scleral epithelia (blue, orange) were found to be similar, while those found at the top, where the sclera had been removed during sample handling, which likely facilitated improved enzyme penetration into the choroid, were different.

Mobility profiles for 2D(2OS) were the most diverse (Figure 4C). Mobility profiles outside both edges of the sclera (orange, blue) were shown to be the most similar, likely as they correspond to similar structures but on different sides of the sclera, while the mobility profile of the scleral stroma (purple) was distinct from these. The remnant of the optic nerve (green) was the most different. The four regions are separated into two zones in PC1: those correlated to the sclera and those to the optic nerve. The scleral regions are then further subdivided across PC2, demonstrating differences in the overarching CS profiles between different regions and further subdivision within these regions. Of the three ions analyzed from CHase ABC-treated sections, 2D(2OS) had the strongest signal and hence the tightest PC groupings (Figure 4Aii,Bii,Cii).

No spatially resolved differences were found for DP2s after CHase ABC treatment (Figure S11A,B), but following treatment with CHase AC, repeatable differences were found for the vitreous humor, protective outer layer, and the sclera, with the vitreous humor and outer protective layer being the most similar (Figure 4D). A similar trend is observed for 2D(1OS), but the vitreous humor and protective layers appear to overlap somewhat, similarly to 3D(0OS) yielded with CHase ABC (Figure 4B), demonstrating reproducibility across different enzymes (Figure 4E). The AC-treated sections appear more diffuse from each other with PCA, despite appearing more similar when compared spectrally. This is due to x-shifts in the mobility axis, caused by changes in buffer gas pressure, occurring during acquisition (Figure S11C). It is unclear why the ABC-treated section does not exhibit the spatial resolution of D(OS). It is possible that delocalization during enzyme treatment or during section preparation, as observed in previous sections, has resulted in homogenization of DP2s across the serial sections (Figure 3A). This is likely, as differences in DP2 profiles between tissue types treated with CHase ABC, which should have different CS profiles, have been observed (Figure S11D). No differences in CHase B-yielded oligosaccharides were observed, suggesting that the DS composition is somewhat homogeneous.

Although the majority of the tissue has a similar oligosaccharide composition, these results are able to distinguish subtle differences in regional intensity, which are no doubt lost during more traditional sample preparations. This represents, for the first time, the ability to monitor alterations of GAG oligosaccharide composition in a spatially resolved manner directly from the tissue.

## Conclusion

The analysis of CS/DS in tissue traditionally includes tissue homogenization and subsequent extraction of CS from whole organs or animals, followed by disaccharide or oligosaccharide analysis involving separation mostly by LC. Such workflows average the oligosaccharide distributions for the entire tissue or organism. Here, we demonstrate that, using MSI coupled with TIMS, we are able to detect, identify, and profile GAG oligosaccharides *in situ* (Figure 2). This includes the spatial localization of mass-separated (either by degree of polymerization or degree of sulfation) oligosaccharides (Figure 3) and – using TIMS profiles – localization of mobility-separated species (Figure 4). The detection and localization of ions with masses and mobilities that correspond to CS disaccharides and oligosaccharides, and DS and HA oligosaccharides (Figures 2 and 3) demonstrate the spatial resolution of multiple GAGs for the first time. This information has never been available before, hence linking it directly to biological function will be the focus of future studies.

The structures within each TIMS peak of the GAG oligosaccharides still need to be elucidated. CID is a poor way to characterize GAG oligosaccharide sequences, as it primarily yields isomeric B and Y fragments and regularly results in sulfate loss.^24^ This is further compounded due to a difficulty in sourcing pure standards that have not been derivatized (i.e., do not have natural mobilities due to linkers utilized for synthesis).

The presence of undersulfated oligosaccharides may be overrepresented due to the MALDI process, however, we do observe highly sulfated oligosaccharides (Figure 2Eiii, Table S1). It also seems that oligosaccharides analyzed from tissue are more heavily sodiated and hence may be more resistant to thermolabile sulfate loss.^35^ Furthermore, if sulfate loss was a significant problem, then the spatial resolution of mass-separated species would not be achievable as shown in Figure 3A,B. We are able to observe distinct tissue regions that correlate to different GAG types and sequences, all of which can be observed in multiple ways through treatment with different enzymes. Changes in ion source either through the use of different matrices which are more sulfate protective or that require less laser power, or by moving to a softer ionization method (for GAGs) such as desorption electrospray ionization (DESI)^36^ may yield a higher quantity of oligosaccharides with a higher DOS. This work in no way claims to accurately portray the entire sulfate composition of CS, DS, and HA in primate retina, but rather demonstrates the ability to release, detect, and profile CS, DS, and HA ions based on mass and mobility from tissue sections in a spatially resolved manner. Further work would be required to accurately and confidently characterize the sulfate composition of this and indeed any other tissue.

## Supporting Information Available

Mobility and tandem mass spectra, assignments for all studied ions, and ion images (PDF)
