# Supplementary material for: Mass Spectrometry Imaging with Trapped Ion Mobility Spectrometry Enables Spatially Resolved Chondroitin, Dermatan, and Hyaluronan Glycosaminoglycan Oligosaccharide Analysis In Situ
Source: Anal Chem. 2024 Oct 30;96(45):17969–77. doi: 10.1021/acs.analchem.4c02706 (PMC11561879; doi:10.1021/acs.analchem.4c02706)
Supplement: Supplementary file 1 — ac4c02706_si_001.pdf [file ac4c02706_si_001.pdf]

Supporting Information: Mass spectrometry imaging with trapped ion mobility spectrometry enables spatially resolved chondroitin, dermatan and hyaluronan glycosaminoglycan oligosaccharide analysis in situ.

Anthony Devlin[*a], Felicia Green[a], Zoltan Takats[a,b].

Affiliations: [a]: The Rosalind Franklin Institute, Harwell Campus, Didcot, OX11 0FA, UK. [b]: Faculty of Medicine, Depart-ment of Metabolism, Digestion and Reproduction, Imperial College London, South Kensington Campus, London, SW7 2AZ, UK

*Corresponding Author: anthony.devlin@rfi.ac.uk.

This document contains supplemental information as pertains to all ions identified as GAGs, their putative assignments, mobilities and subsequent spatial localisation in primate retinae sections.

Table of contents:

Page S3: Table of all CS/DS/HA ions located *in situ*.

Pages S4:7: **Extracted ion mobilities.**

- Page S4: EIM of DP2 ions.
- Page S5: EIM of DP4 ions.
- Page S6: EIMs of DP6 and unsulphated DP4-6 ions.
- Page S7: Mobility and MS/MS analysis of GAG anomers.

Pages S8:12: **Histology/Ion images.**

- Page S8: H&E stained and optical images of CHase ABC, AC and B treated sections.
- Page S9/10: Ion images of CHase ABC treated sections.
- Page S11: Ion images of CHase AC treated sections
- Page S12: Ion images of CHase B treated sections

Page S13: DP2 analysis of CHase ABC treated sections.

Page S14: References

All masses that correspond to GAG ions are located in **table S1**. During long imaging experiments, the mass calibration of the TOF can drift and, since no lock-mass calibration was used in these experiments, this is likely why all ppm errors are negative. The 2D(3OS)+2Na ion has an unusually high ppm (-17.81) error compared to the others (average = -6.48 ppm). The large error is likely due to low signal intensity (this can be observed with the isobaric noise appearing in the mobilities in **Fig.2Eiii**, orange arrow, which is affecting the peak centroid and therefore the mass accuracy The ion has a comparable TIMS profile to those of the 2D(3OS)+2Na standards (**Fig 2Eiii**), is only present in samples treated with CHase AC and can be localised to tissue specific regions (**Fig. S9Bviii**). Furthermore, it forms part of a Na adduct series, as many of the other GAG ions do due to the high propensity of GAGs to bind alkali metal ions[1], and has been observed in other tissues treated with CHase AC with sufficient signal intensity to perform CID and yield assignable fragments (data not shown). Hence, we are confident that it is a 2D(3OS)+Na Ion, despite the larger mass error than other ions.

Table S1: Sulphated saccharide ions detected from primate retinae after CHase digestion and their assignments. Ions were assigned based on appearance after CHase digestion, accurate mass and mobility compared to standards. D(1-2OS), 2D(0-2OS) and 3D(0-2OS) were further characterised with MS/MS (**Fig.1B**) *Observed with limited signal intensity.

Observed Mass / m/z	Theoretical mass / m/z	Error / ppm	Assignment	Ion	Enzyme
458.058	458.060	-6.23	D(OS)	$[M-H]^-$	ABC, AC, B
480.039	480.042	-6.46	D(OS)	$[M+Na-2H]^-$	ABC, AC, B
496.012	496.016	-8.34	D(OS)	$[M+K-2H]^-$	ABC, AC
559.996	559.999	-5.92	D(2OS)	$[M+Na-2H]^-$	ABC, AC, B
581.978	581.981	-5.78	D(2OS)	$[M+2Na-3H]^-$	ABC, AC
757.210	757.215	-6.34	2D(0OS)	$[M-H]^-$	ABC,AC
779.192	779.197	-7.12	2D(0OS)	$[M+Na-2H]^-$	ABC,AC
837.165	837.172	-7.91	2D(1OS)	$[M-H]^-$	ABC,AC, B
859.148	859.154	-7.18	2D(1OS)	$[M+Na-2H]^-$	AC
881.130	881.136	-6.48	2D(1OS)	$[M+2Na-3H]^-$	AC
939.104	939.111	-6.69	2D(2OS)	$[M+Na-2H]^-$	ABC*, AC
961.086	961.093	-7.31	2D(2OS)	$[M+2Na-3H]^-$	AC
983.068	983.075	-7.19	2D(2OS)	$[M+3Na-4H]^-$	ABC, AC, B
1041.031	1041.049	-17.81	2D(3OS)	$[M+2Na-3H]^-$	AC
1063.024	1063.031	-6.57	2D(3OS)	$[M+3Na-4H]^-$	AC
1085.006	1085.013	-6.76	2D(3OS)	$[M+4Na-5H]^-$	AC
1180.284	1180.290	-5.72	3D(0OS)	$[M+2Na-3H]^-$	ABC*, AC
1216.276	1216.283	-5.74	3D(1OS)	$[M-H]^-$	ABC*, AC
1238.257	1238.265	-6.48	3D(1OS)	$[M+Na-2H]^-$	ABC*, AC
1318.215	1318.222	-5.72	3D(2OS)	$[M+Na-2H]^-$	ABC*, AC
1340.198	1340.204	-4.84	3D(2OS)	$[M+2Na-3H]^-$	AC
1362.178	1362.186	-5.68	3D(2OS)	$[M+3Na-4H]^-$	AC
1384.161	1384.168	-4.83	3D(2OS)	$[M+4Na-5H]^-$	AC
1420.150	1420.161	-7.75	3D(3OS)	$[M+2Na-3H]^-$	AC
1442.136	1442.143	-4.89	3D(3OS)	$[M+3Na-4H]^-$	AC
1464.125	1464.118	-4.92	3D(3OS)	$[M+4Na-5H]^-$	AC
1486.101	1486.107	-3.93	3D(3OS)	$[M+5Na-6H]^-$	AC
1566.064	1566.057	-4.00	3D(4OS)	$[M+5Na-6H]^-$	AC
1588.040	1588.046	-3.21	3D(4OS)	$[M+6Na-7H]^-$	AC

Extracted Ion Mobilities

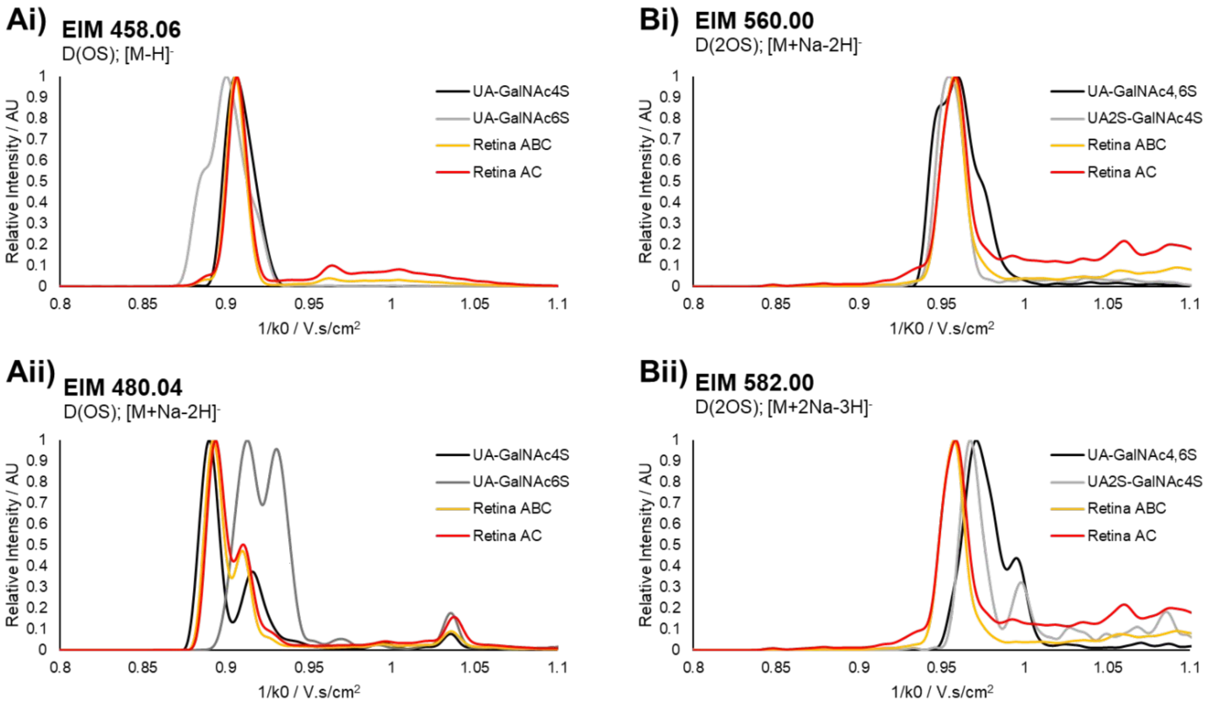

Figure S1: EIM of DP2 CS ions detected from ape retinae compared to CS ions from shark cartilage. **A)** D(OS), **B)** D(2OS)

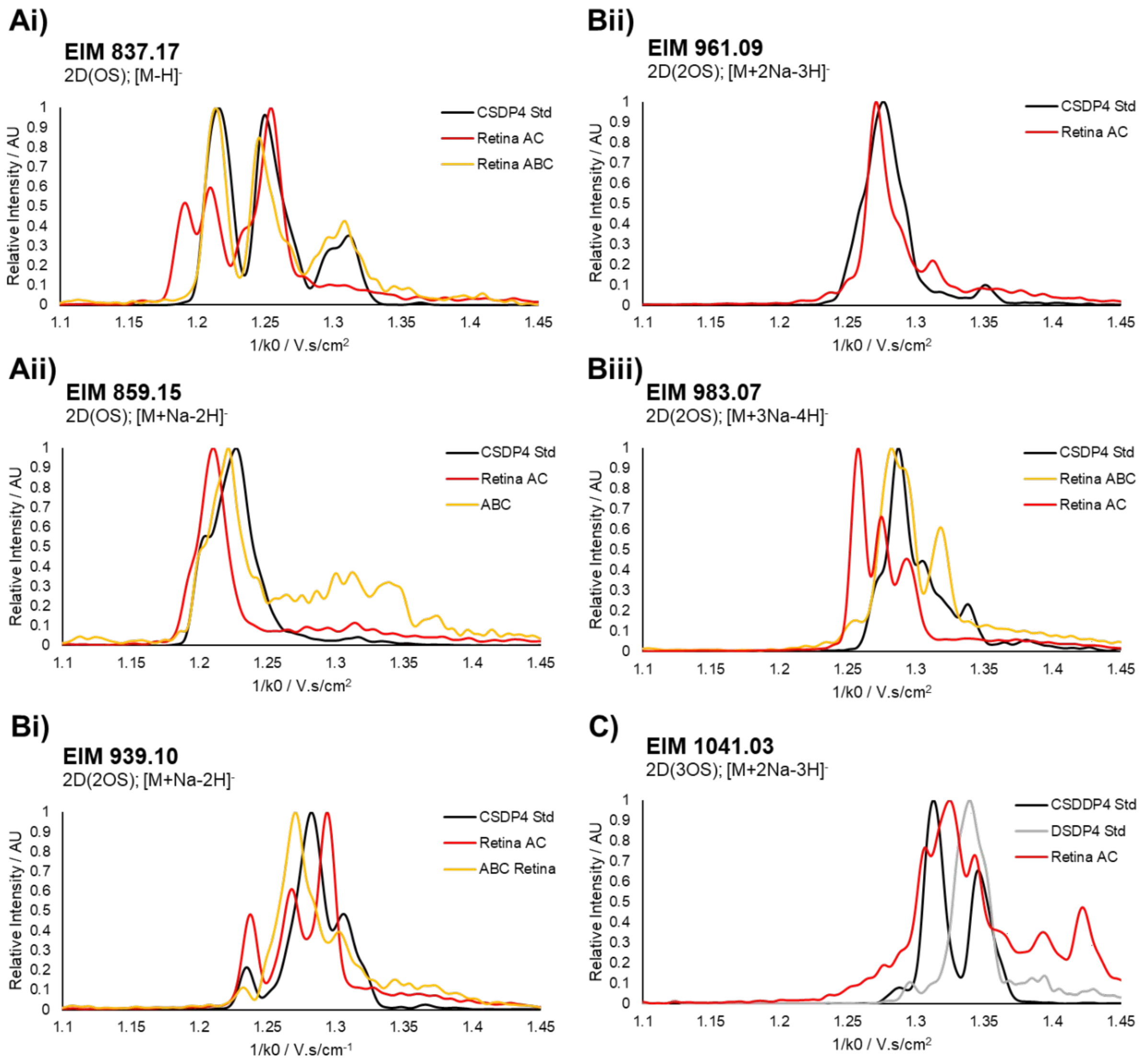

Figure S2: EIM of sulphated DP4 CS ions detected from ape retinae compared to CS ions from shark cartilage. **A)** 2D(OS), **B)** 2D(2OS). **C)** 2D(3OS)

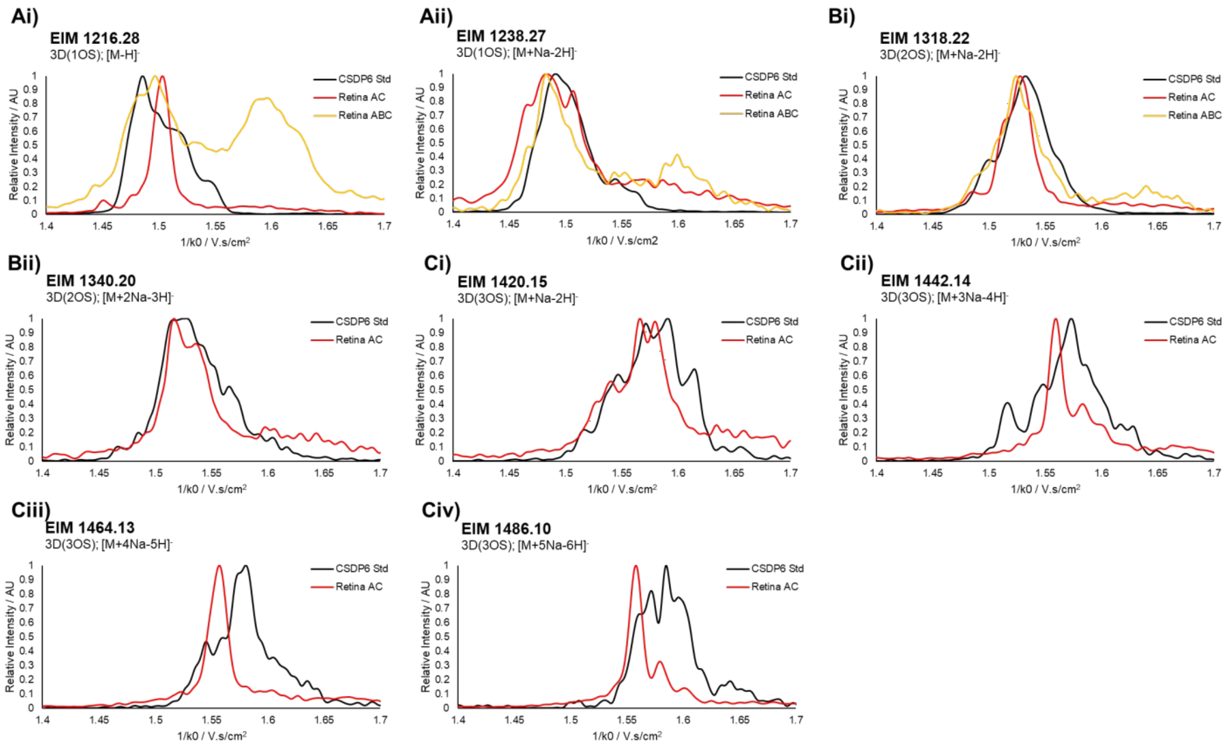

Figure S3: EIM of sulphated DP6 CS ions detected from ape retinae compared to CS ions from shark cartilage. **A)** 3D(OS), (**I**, ii), second peak for ABC treated sections are likely overrepresented isobaric noise due to low signal strength. **B)** 3D(2OS). **C)** 3D(3OS)

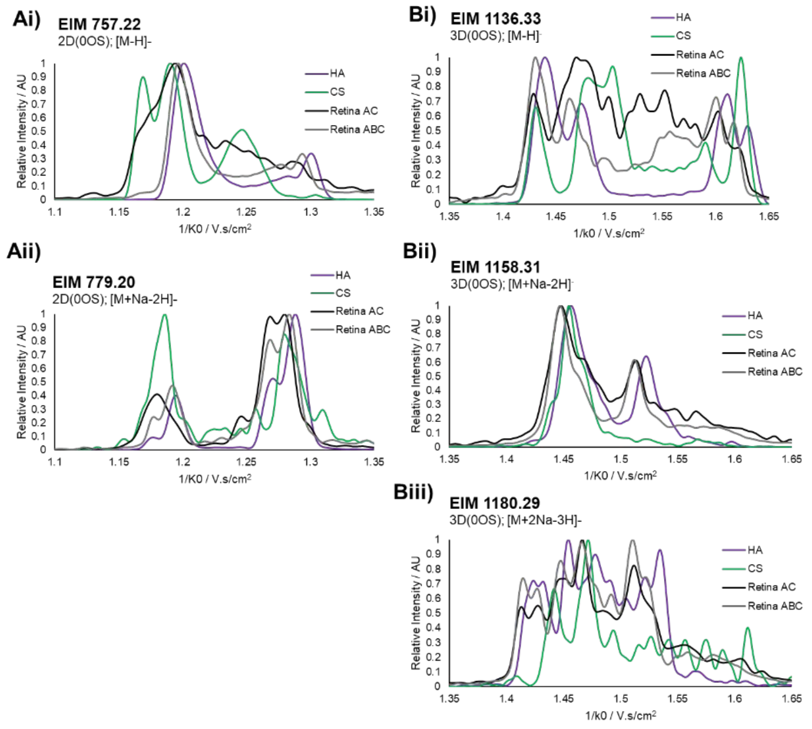

Figure S4: EIM of unsulphated CS/HA ions detected from ape retinae compared to CS ions from shark cartilage and HA ions. **A)** DP4 **B)** DP6.

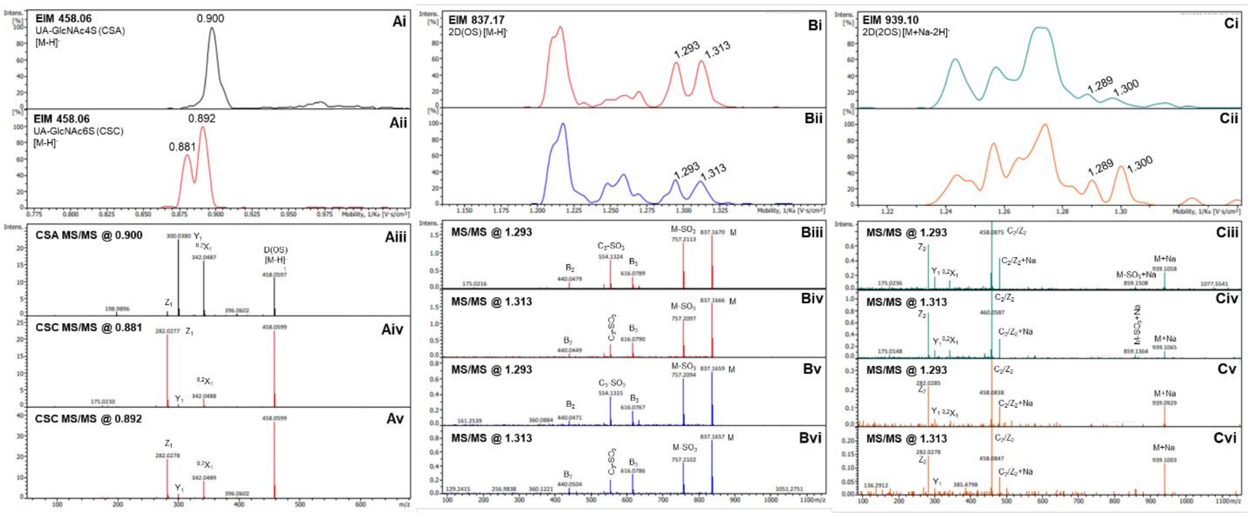

Figure S5: MS/MS detection of anomers in DP2s and DP4s. **A)** Pure CSC standard, EIM of 458.06 ([M-H]$^-$), has multiple peaks (**ii**). CID of each peak yields similar MS/MS spectra (**iv, v**) suggesting that the same sequence is present. **B)** Analysis of DP4, 2D(OS) from two different CS polymers. In EIM of 837.17 ([M-H]$^-$), the final two peaks are baseline resolved (**i, ii**) but have comparable fragmentation both intra- and inter- sample (**iii:vi**), suggesting the presence of anomers. **C)** Analysis of DP4, 2D(2OS) from two different CS polymers. In EIM of 939.10 ([M+Na-2H]$^-$), the final two peaks are resolved (**i, ii**) but have comparable fragmentation both intra- and inter- sample (**iii:vi**), suggesting the presence of anomers.

Histology/Ion images

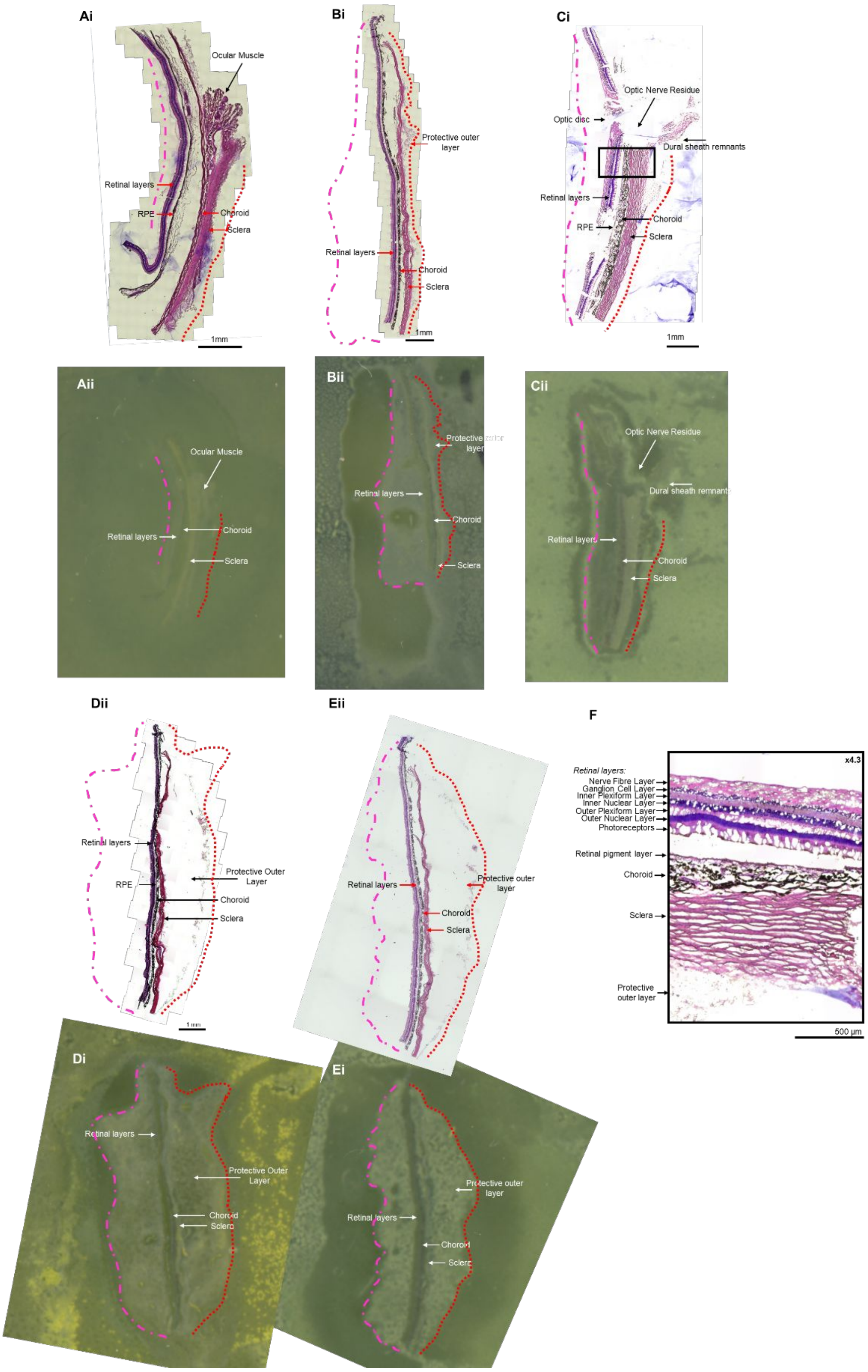

Figure S6: Retina histology of the sections studied. **i**) H&E stained sections, performed after MALDI, following washing in 100% methanol to remove matrix. **ii**) optical images of sections after matrix application, demonstrating the edges of the protective outer layer and the vitreous humor. **A)** ABC treated section (**Fig. 5**). **B)** AC treated section (**Fig.5**). **C)** ABC treated section (**Fig. 6**). **D)** AC treated section (**Fig. 6**). **E)** CHase B treated section (**Fig. S15**). **F**) zoom in of (**A**) (black box), with labelled

retinal layers. Red dotted lines indicate the edge of the protective outer layer and pink hashed lines indicate the edge of the vitreous humor.

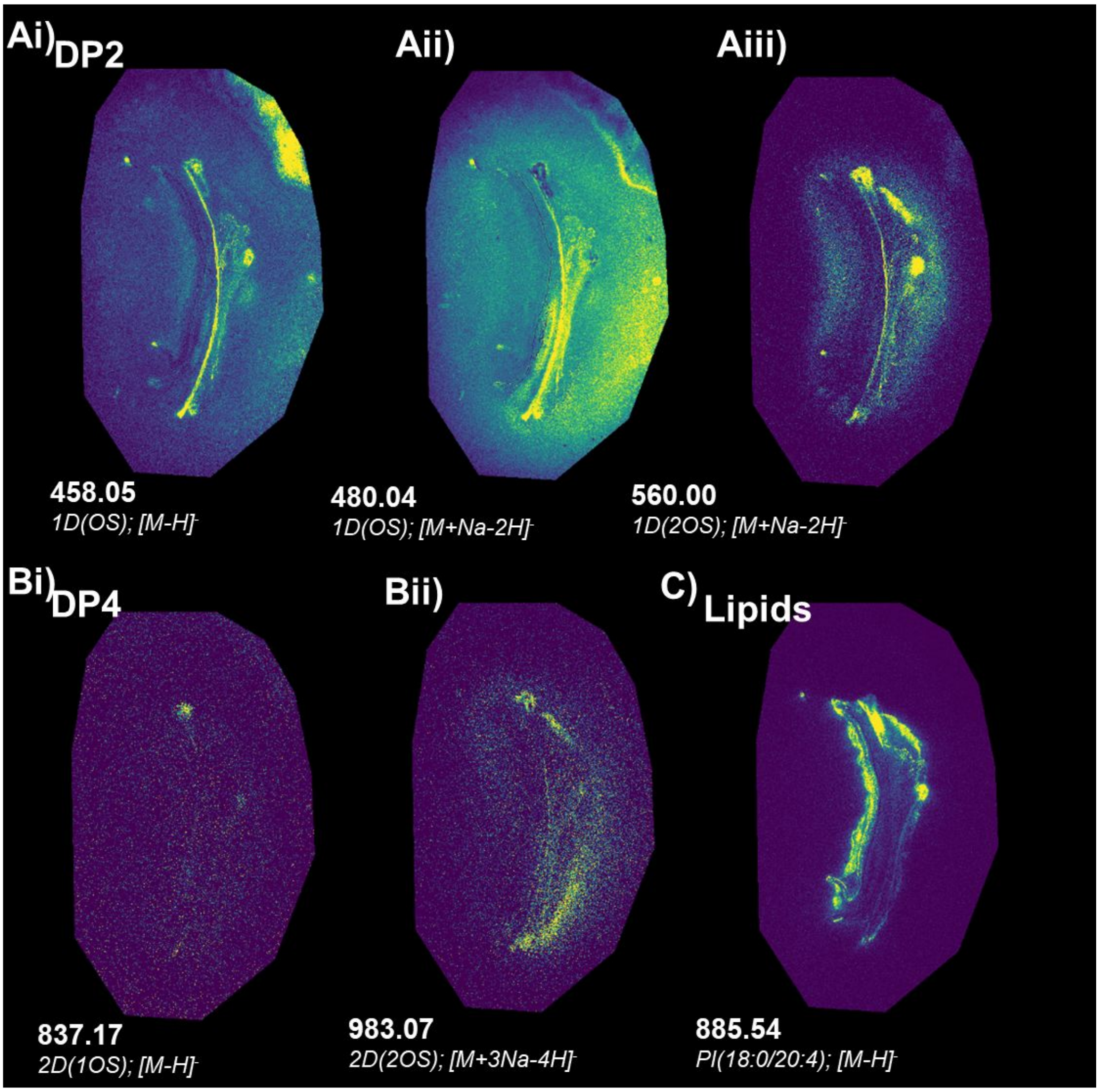

Figure S7: Ion images for each GAG ion found *in situ* for CHase ABC treated sections. **A)** DP4 **B)** DP6. (Used in **Fig. 4**). **C)** PI(18:0/20:4). The image was acquired over a mass range of 50-1100.

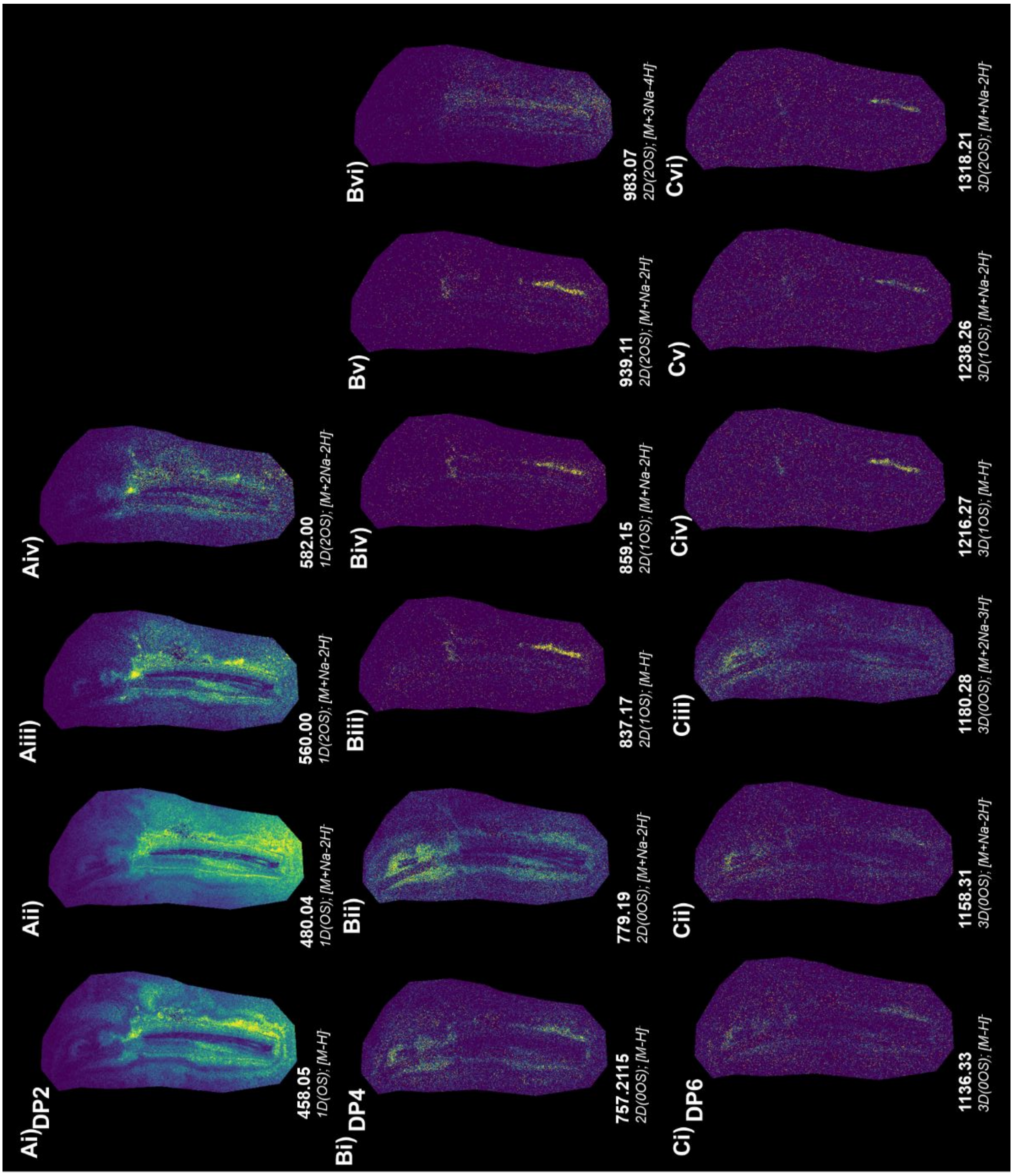

Figure S8: Ion images for DP2-6s after CHase ABC digestion (used in **Figs. 1 and 5**). **A)** DP2s. **B)** DP4s. **C)** DP6s. The image(s) were acquired over a mass range of 50-2000, enabling detection of DP6s. Poor signal strength and some delocalisation are observed, likely due to sample degradation during storage. 1 of 3 repeats are displayed here.

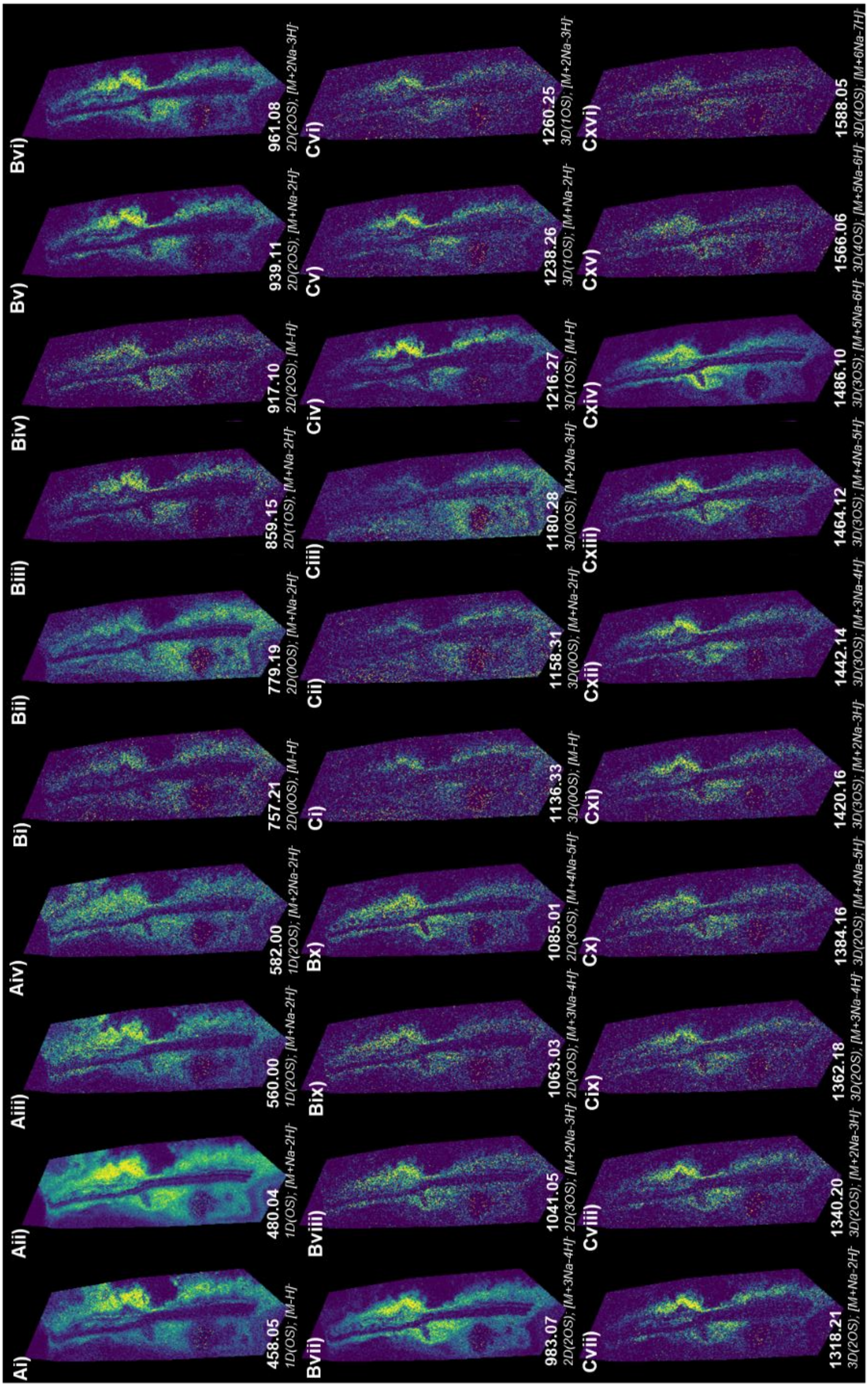

Figure S9: **I**on images for **A)** DP2, **B)** DP4 and **C)** DP6 ions after CHase AC digestion (used in **Figs. 1, 3 and 4**)

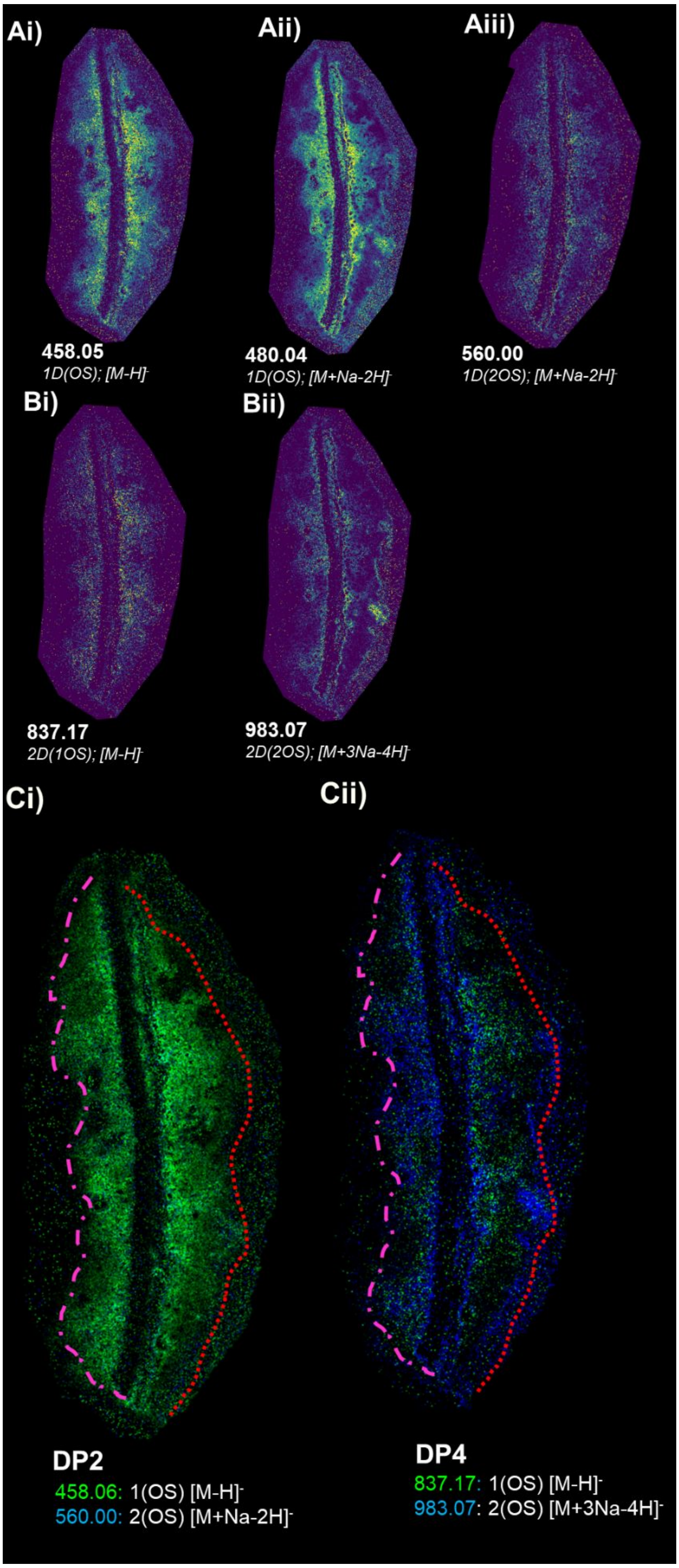

Figure S10: Ion images for DP2-4s after CHase B digestion. **A**) DP2s. **B**) DP4s. **C**) stacked ion images. Red dotted and pink hashed lines indicate the edge of the outer protective layer and the vitreous humor respectively.

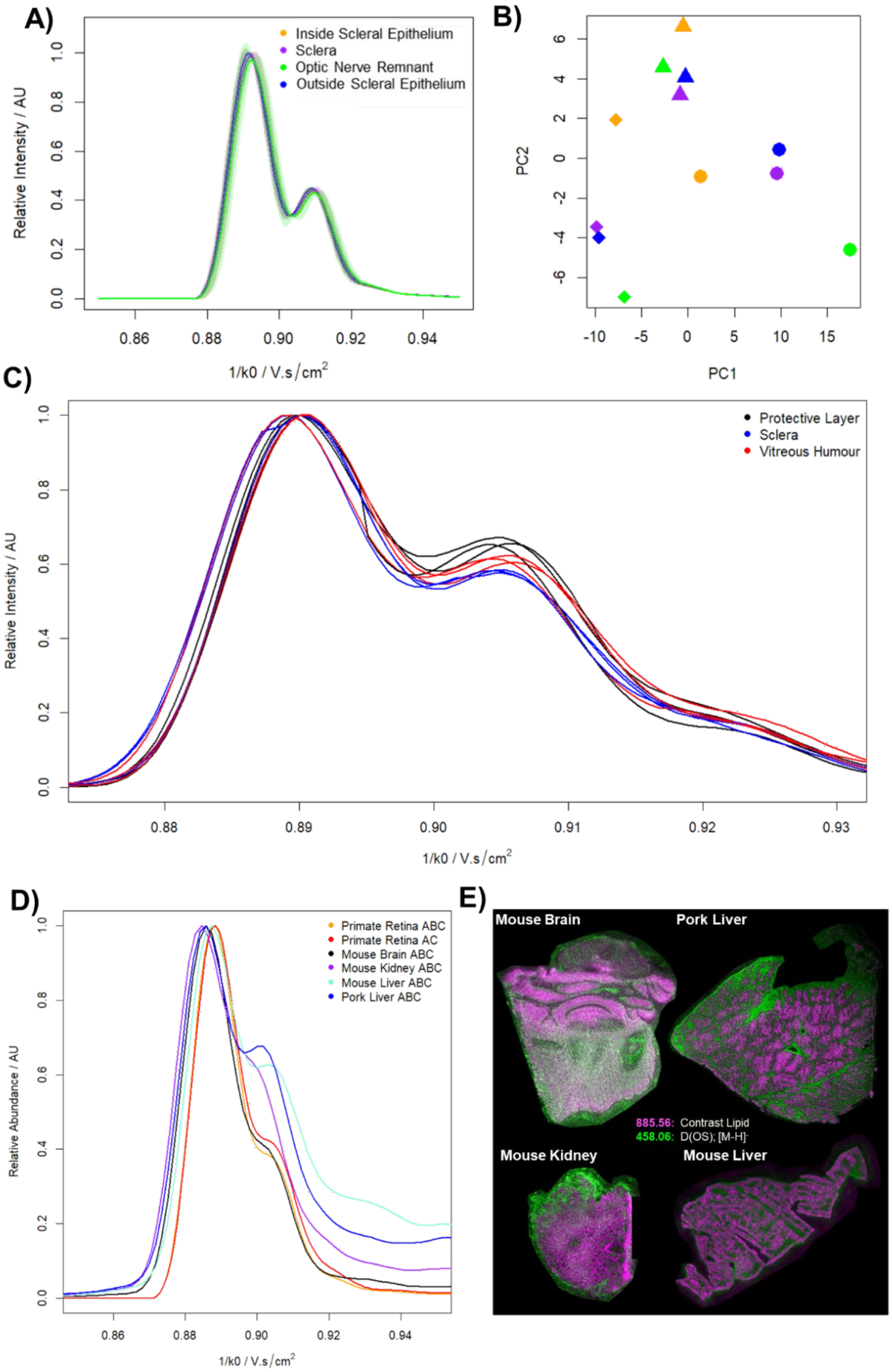

Figure S11: Spatially resolved DP2 analysis. **A)** Spatially resolved mobilities for EIM 480.04. **B)** PCA of EIMs. **C)** EIMs of 480.04; D(OS); $[M+Na-2H]^-$ for CHase AC treated sections, exhibiting mobility axis shifts. **D)** EIMs for 480.04 for different tissues, organisms and enzymes. **E)** Ion images for D(OS) in the tissues.

References

(1) Pepi, L. E.; Sanderson, P.; Stickney, M.; Amster, I. J. Developments in Mass Spectrometry for Glycosaminoglycan Analysis: A Review. *Mol. Cell. Proteomics MCP* **2021**, *20*, 100025. https://doi.org/10.1074/mcp.R120.002267.
